# Multiple sclerosis drug FTY-720 toxicity is mediated by the heterotypic fusion of organelles in neuroendocrine cells

**DOI:** 10.1038/s41598-019-55106-w

**Published:** 2019-12-05

**Authors:** Yolanda Gimenez-Molina, Virginia García-Martínez, José Villanueva, Bazbek Davletov, Luis M. Gutiérrez

**Affiliations:** 1 Instituto de Neurociencias de Alicante, Universidad Miguel Hernández-Consejo Superior de Investigaciones Científicas, Sant Joan d’Alacant, Alicante 03550 Spain; 2 Department of Biomedical Science, University of Sheffield, Firth Court, Western Bank, Sheffield, S10 2TN UK

**Keywords:** Cellular neuroscience, Molecular medicine

## Abstract

FTY-720 (Fingolimod) was one of the first compounds authorized for the treatment of multiple sclerosis. Among its other activities, this sphingosine analogue enhances exocytosis in neuroendocrine chromaffin cells, altering the quantal release of catecholamines. Surprisingly, the size of chromaffin granules is reduced within few minutes of treatment, a process that is paralleled by the homotypic fusion of granules and their heterotypic fusion with mitochondria, as witnessed by dynamic confocal and TIRF microscopy. Electron microscopy studies support these observations, revealing the fusion of several vesicles with individual mitochondria to form large, round mixed organelles. This cross-fusion is SNARE-dependent, being partially prevented by the expression of an inactive form of SNAP-25. Fused mitochondria exhibit an altered redox potential, which dramatically enhances cell death. Therefore, the cross-fusion of intracellular organelles appears to be a new mechanism to be borne in mind when considering the effect of FTY-720 on the survival of neuroendocrine cells.

## Introduction

The exocytotic fusion of vesicles with the plasma membrane is a fundamental event in neuronal and endocrine systems, facilitating the release of active substances like neurotransmitters and hormones. This is a sequential process that initially involves vesicle transport to the membrane mediated by cytoskeletal proteins^1,2^, their docking at the membrane and finally, their fusion and the release of their cargo (e.g., neurotransmitters) through the specific interaction of SNARE proteins (soluble N-ethylmaleimide sensitive factor attachment protein receptor), and of the lipids that constitute the vesicular and plasma membrane^3,4^.

The relatively passive role of lipids assumed in the traditional model of exocytosis that is based on the catalytic activity of fusogenic proteins has evolved in recent times, whereby lipids are now considered to be more active elements in neurosecretion^5–7^. In this regard, it is important to note the role of signalling lipids released by the activity of phospholipases as direct modulators of the SNARE fusion machinery, over and above the role of structural lipids. For example, arachidonic acid (AA) released from phospholipid membranes upregulates syntaxin-1 promoting the incorporation of this protein into SNARE complexes^8,9^. Moreover, the sphingolipid backbone that may be released, sphingosine, interacts with the vesicular SNARE synaptobrevin to enhance the formation of the exocytotic fusion complex, thereby enhancing the release of neurotransmitters and hormones^10,11^.

Interestingly, the sphingomimetic drug FTY-720 (Fingolimod), a drug approved for the treatment of multiple sclerosis (MS)^12^, also increased the formation of the ternary SNARE complex and augmented neuroendocrine secretion^13^. Indeed, more evidence appeared in recent years suggesting that this compound accumulates in cells of the CNS, modulating the activity of neurons and astrocytes^14,15^, potentially making it suitable to treat a variety of neurological syndromes like ischemia, stroke and neurodegeneration^16–18^. Furthermore, this drug has been also proposed for the treatment of neuronal cancers given its potential to induce the death of neuroblastoma cells^19^.

In view of the potential of this drug to interfere with multiple molecular mechansims associated with a variety of syndromes, we further studied the possible effects and targets of FTY-720 on the behaviour of chromaffin cells, a well established exocytotic neuroendocrine model^1,20,21^. Our results show that FTY-720 not only affects secretory process but also, it induces the homotypic and heterotypic fusion of organelles in the cytosol, impairing mitochondrial function and provoking cell death.

## Results

### FTY-720 affects the secretory response in bovine chromaffin cells in culture

**Figure 1:**
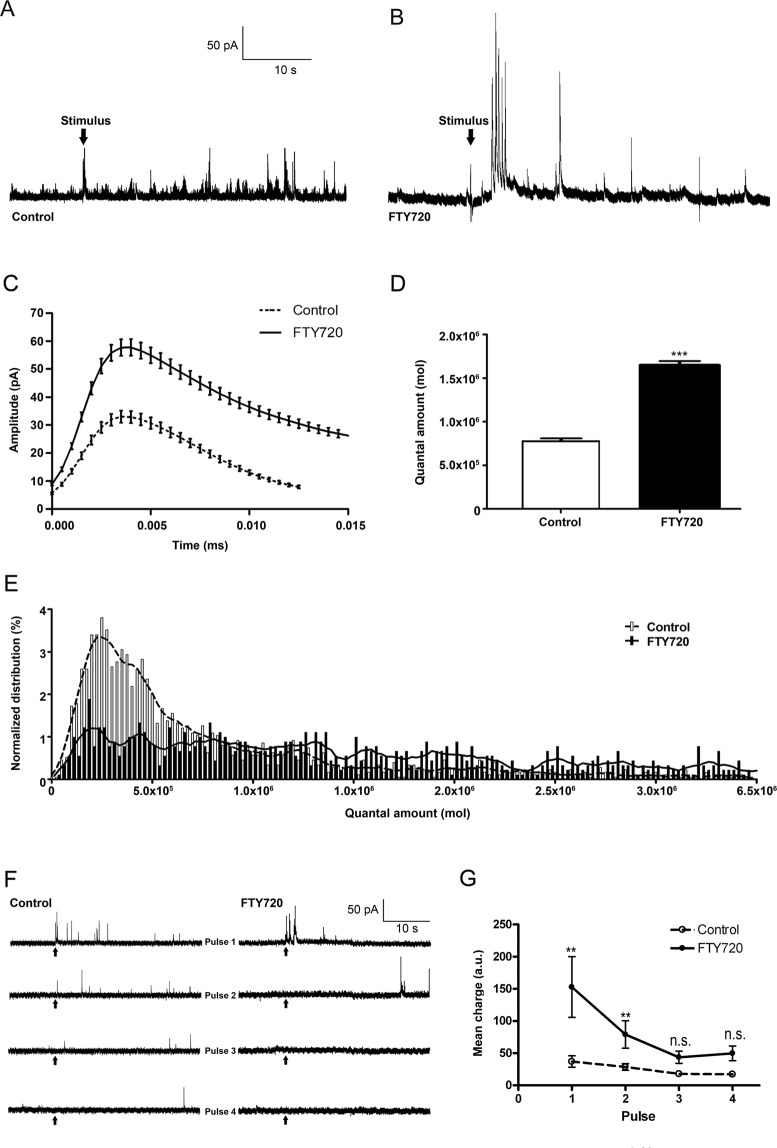
Secretory responses of chromaffin cells exposed to FTY-720. (**A,B)** Amperometric measurements from cultured bovine chromaffin cells stimulated by depolarization with a 59 mM KCl solution: control cells (**A**) and cells treated with a 20 µM FTY-720 for 15 mins (**B**). (**C**) Averaged amperometric fusion events and parameters from control (N = 1262 events) and FTY-720-treated cells (N = 901 events). (**D**) FTY-720 enhances the quantal amount of catecholamines released per single event. (**E**) Distribution of the quantal events in control and FTY-720 treated cells. (**F**) Example of amperometrical responses to cell depolarization in repetitive 10 s pulses. (**G**) Mean secretory responses obtained by amperometry trace integration during each pulse (charge) in both control and FTY-treated cells.Statistical significance was assessed with a Mann-Whitney U-Test: **P ≤ 0.005. ***P ≤ 0.0001.

Among the drugs derived from the structure of signalling lipids, FTY-720 is an analogue of sphingosine that has been used extensively to produce immunosuppression^22^ and as such, it has been approved for the treatment of MS^12^. As a result, we were prompted to study whether, in addition to its immune mechanisms, this compound also affects exocytosis in a similar manner to signalling lipids^10,11,23,24^. To test this, chromaffin cells in culture were stimulated by depolarization with a high KCl buffer and their secretory activity was studied by amperometry^25,26^. The number and frequency of vesicular fusions were analysed in amperometric recordings from cells before and after treatment with FTY-720 (20 µM) for 15 min, an optimal concentration that enhances SNARE complex formation (Fig. 1B)^13^. FTY-720 provoked a characteristic enhancement in the number of secretory events in cells, first because basal fusion was observed in the absence of stimulation and second, because the secretory events elicited by cell depolarization are clearly enhanced relative to untreated control cells (Fig. 1A,B). In addition, FTY-720 also seemed to increase the amplitude of the individual amperometric peaks when analysed using the Quanta Analysis program^27^. After averaging 900 or more events from individual cells in at least 3 different cultures (Fig. 1C), it was clear that treatment with FTY-720 provoked a 2.5 fold enhancement in the quantal amount of catecholamines released per individual event (charge obtained from peak integration, Q value in molecules per event) relative to control untreated cells (Fig. 1D).

The spread in the distribution indicated that secretory events occurred in which less vesicular content was released and those with a much larger quantal content, characterized by prolonged opening of the fusion pore (Fig. 1E). Therefore, FTY-720 enhanced single vesicle fusion with functional characteristics resembling that produced by its physiological counterparts, AA and sphingosine^23,24^. Accordingly, treatment with this drug means that cell stimulation produces more fusion events, and it enhances quantal secretion by provoking the release of microvesicles as well as vesicles with higher content. Indeed, the average release was dominated by this latter effect causing an enhancement in the vesicular content released per event.

Interestingly, the release of vesicles was clearly affected by repetitive stimulations, which reflected an impairment of both control and FTY-720 treated cells to recruit new vesicles (examples in Fig. 1F). Averaging these responses after trace integration, showed a clear decrease in the mean charge released in the second and sequent pulses in relation with the initial one in both control and FTY-treated cells (Fig. 1G). In fact, the mean charge released obtained during the 3^rd^ and 4^th^ pulses was similar in both experimental conditions (n.s. values, Fig. 1G). This impairment in vesicle recruitment is related to the reduced vesicle and F-actin mobility induced by FTY-720, as witnessed by confocal fluorescence microscopy (data not shown) and consistent with earlier data from astrocytes^28^.

### Confocal microscopy reveals that FTY-720 provokes a reduction in the number of chromaffin granules and concomitant changes in the aspect of the mitochondria

**Figure 2:**
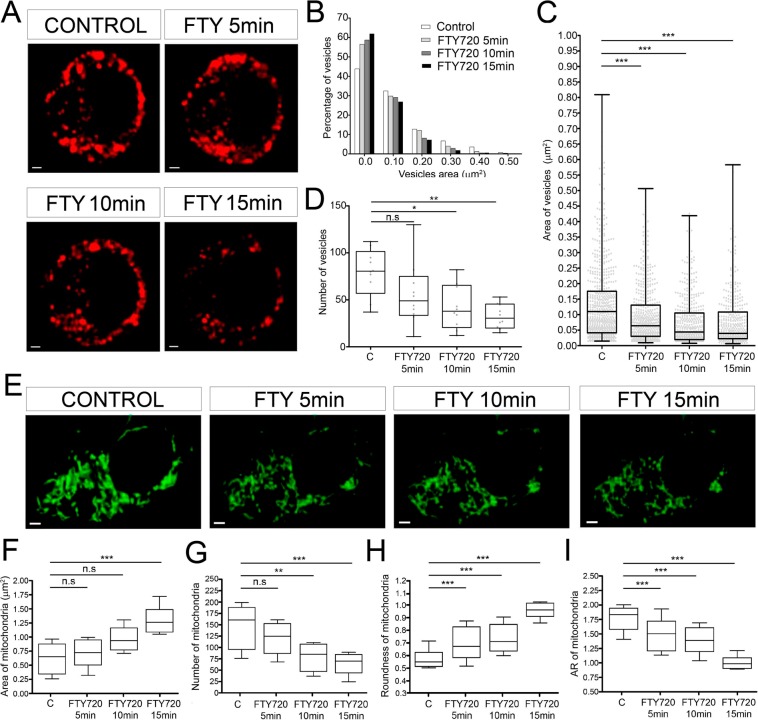
Morphometric changes of vesicles and mitochondria in control and FTY-720 treated cells. (**A**) Time-lapse confocal fluorescence microscopy of a representative cultured chromaffin cell expressing RFP-NPY (red) to show vesicles from control and FTY-720 treated cells (5, 10 and 15 min). (**B**) Vesicular size frequency distribution comparing all conditions (**C**; FTY720 5,10 and 15 min) by size. (**C,D**) Box and whiskers and scatter plot vertical graphs of vesicle area (**C**) and number (**D**) by condition (N = 10 cells; N_control_ = 686, N_FTY720 5min_ = 554, N_FTY720 10min_ = 419, N_FTY720 15min_ = 393 vesicles). **(E**) Time-lapse confocal fluorescence microscopy images from a representative live cultured chromaffin cell labeled with MitoTracker green to identify the mitochondria in control and FTY-720 treated cells (5, 10 and 15 min). (**F–I**) Box and whiskers vertical graphs of the mitochondrial area (**F**), number (**G**), roundness (**H**) and aspect ratio (**I**) per condition (N = 5 cells: N_control_ = 804, N_FTY720 5min_ = 621, N_FTY720 10min_ = 424, N_FTY720 15min_ = 350 mitochondria). Box and whiskers graph shows the mean as the central box line, the SEM as the box limits (top and bottom) and the amplitude variability through the whiskers (box emergent lines). Statistical significance was assessed by a Kruskal-Wallis Test: n.s., non-significant (P ≥ 0.05), *P ≤ 0.05, **P ≤ 0.01, ***P ≤ 0.001. Scale bars represent 1 μm.

Changes in the vesicular content released following treatment with FTY-720 could be due to altered chromaffin granule size or content and therefore, we studied this possibility using fluorescence confocal microscopy of chromaffin cells expressing the vesicular marker NPY, as indicated elsewhere^29^. Interestingly, after a 10–15 min treatment with FTY-720 (20 µM) there was a reduction in the number of the vesicles labelled with mRFP coupled NPY (red fluorescence, Fig. 2). Measuring the average number of vesicles in cells showed there was a significant reduction after as little as 5 min in the presence of FTY-720, reaching a 30–40% decrease after a 10–15 min treatment. In addition, there was a decrease in the average size of the granules following exposure to FTY-720, which could again be detected as soon as 5 min after incubation with this drug, and that reached a 30% decrease in area (50% reduction in volume) after a 10 min treatment. Consequently, we detected an early change in the formation of microvesicles following incubation with FTY-720 and a concomitant decrease in the number of vesicles 15 min later. These alterations would support the appearance of microvesicle fusions, yet they would appear to contradict the overall increase in the size of the vesicular content released per event. However, it is important to bear in mind the simultaneous change in the mitochondrial structure evident following Mitotracker green labelling (Fig. 2E), associated with an increase in mitochondrial size (Fig. 2F) and a decrease in their number (Fig. 2G). In addition, these organelles shifted from their characteristic elongated form to a shortened and somewhat rounded appearance after a 15 min treatment with FTY720. This observation was supported by an increase in the mitochondria’s roundness (Fig. 2H) and a decrease in their Aspect Ratio (AR, ratio of the longer axis over the shorter one: Fig. 2I). Hence, FTY-720 provokes a clear change in the morphology of the mitochondria that parallels the decrease in the number of chromaffin vesicles.

### TIRF and confocal microscopy detect homotypic and heterotypic fusion of organelles following FTY-720 treatment

**Figure 3:**
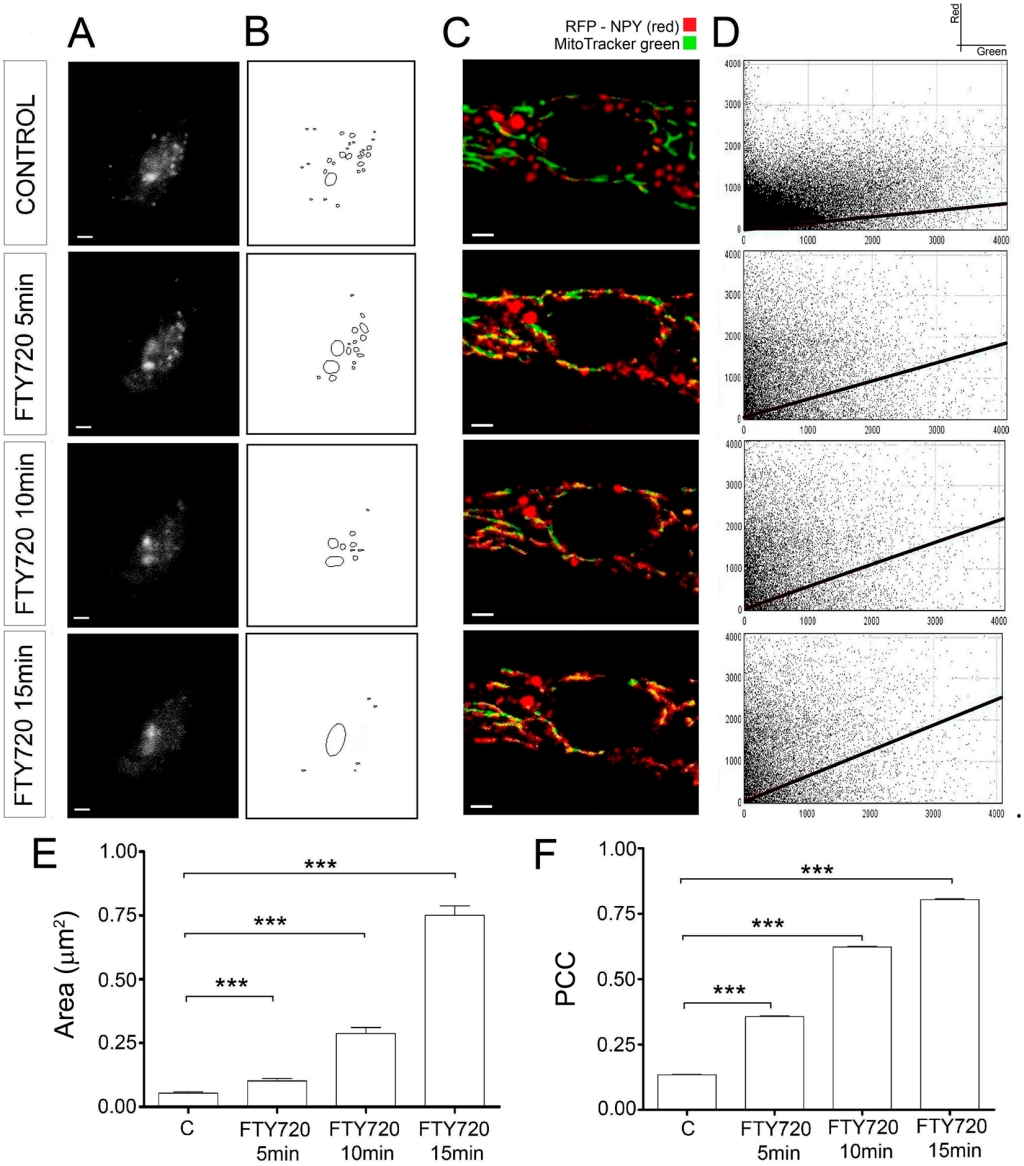
Colocalization of organelles during FTY-720 treatment. (**A**) Time-lapse TIRF microscopy images from a representative cultured chromaffin cell expressing RFP-NPY (red) to detect vesicles at the plasma membrane of control and FTY-720 treated cells (5, 10 and 15 min). (**B**) Vesicular masks at plasma membrane in each condition. (**C**) Time-lapse confocal fluorescence images from a representative live cultured chromaffin cell labelled with MitoTracker green to visualize mitochondria, and expressing RFP-NPY (red) to detect vesicles in control and FTY-720 treated cells (5, 10 and 15 min). (**D**) Representative cytofluorograms per condition (Y axis for red fluorescence and X axis for green fluorescence). The black lines indicate the fluorescence colocalization as a direct correlation of the line’s slopes. (**E**) Mean ± SEM values of the cortical vesicular mask area per condition (N = 5 cells: N_control_ = 106, N_FTY720 5min_ = 59, N_FTY720 10min_ = 37, N_FTY720 15min_ = 19 masks). (**F**) Mean ± SEM values of the Pearson’s Correlation Coefficient per condition (N = 5 cells: N = 10 points/cell). Statistical significance was assessed with a Kruskal-Wallis Test: ***P ≤ 0.001. Scale bars represent 1 μm.

The mitochondrial and vesicle populations are particularly dense in the cortical area of chromaffin cells in culture^30,31^ and thus, changes in the destiny of vesicles in the sub-plasmalemma region (up to 150–200 nm from the cell membrane) were tracked by total internal reflection fluorescent microscopy (TIRFM)^32,33^. During the initial 5–10 min after exposure to FTY-720, NPY-mRFP labelled vesicles were apparently smaller in size but later, these fluorescent organelles appeared to become more elongated and larger (Fig. 3A,B). These latter changes were accompanied by a dramatic reduction in the number of vesicles observed, consistent with the confocal microscopy data and with the increase in the size of the labelled organelles measured by mean area determination (Fig. 3E). Consequently, TIRFM experiments revealed an intriguing change in the properties of vesicles, with an initially shift towards the formation of microvesicles followed by their apparent fusion, both homotypic fusion or heterotypic fusion with other major cortical membrane structures, which was reflected in a reduction in the number of vesicles observed under evanescent field and in the formation of elongated structures.

Evidence of cross-fusion between chromaffin granules and mitochondria (heterotypic fusion) was obtained by analysing the co-localization of the NPY-mRFP vesicular labelling (red fluorescence in Fig. 3C) and that of mitotracker green in mitochondria (green fluorescence in Fig. 3C) in sequential confocal microscopy images. FTY-720 appears to induce cross-fusion of these organelles as their fluorescent signals appear to co-localize, detected as a yellow colour in the merged images (Fig. 3C). This co-localization was further supported by the Pearson coefficient that correlated the distribution of the red and green fluorescence signals (Fig. 3D), with enhanced co-localization as the FTY-720 treatment increases, shifting the Pearson coefficient from 0.12 in the absence of FTY-720 to 0.36, 0.58 and 0.70 after a 5, 10 and 15 min treatment with FTY-720 (Fig. 3F).

### Electron microscopy confirmed the alterations to chromaffin granules, and the homo and heterotypic fusion of organelles

**Figure 4:**
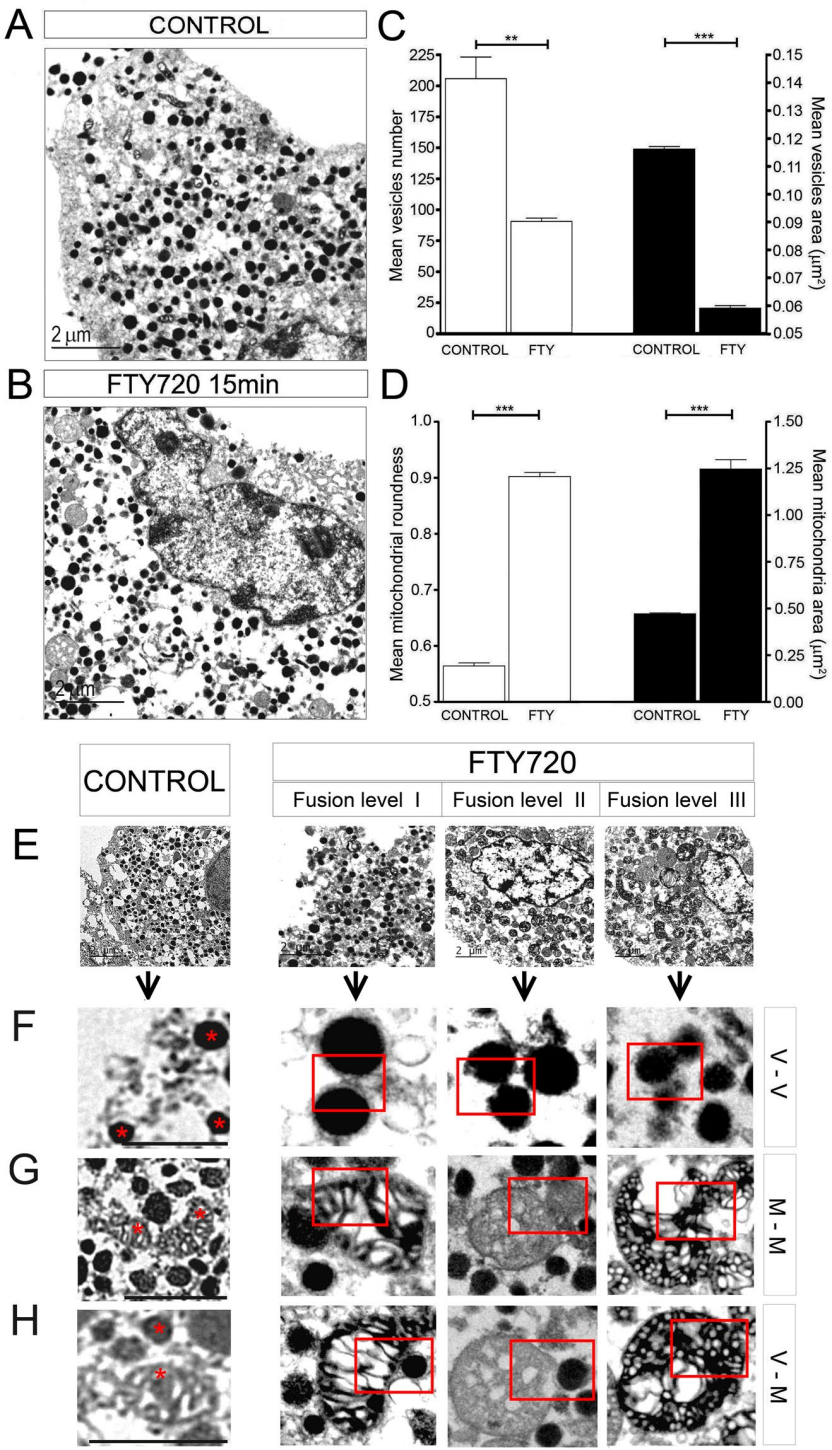
Electron microscopy shows organelle transformation mediated by FTY-720 induced fusions. (**A,B**) EM micrographs of representative cultured control chromaffin cells (**A**), and after incubation with FTY-720 for 15 min (**B**). (**C**) Mean ± SEM vesicle number (white bars) and area (black bars) per condition (N_control_ = 6 cells, 1235 vesicles; N_FTY720 15min_ = 6 cells, 542 vesicles). (**D**) Mean ± SEM values of mitochondrial roundness (white bars) and area (black bars) per condition (N_control_ = 6 cells, 932 mitochondria; N_FTY720 15min_ = 6 cells, 406 mitochondria). **(E**) EM micrographs from cultured control chromaffin cells and after FTY-720 (15 min) showing different fusion levels (I to III) after treatment. (**F–H**) Amplified images of representative cytoplasmic sections including examples of all types of fusion (rows) and levels (columns; I to III) between vesicles (V), and mitochondria (M). Red stars and boxes indicate interaction between elements. Statistical significance was assessed with a Mann-Whitney U-Test: **P ≤ 0.01, ***P ≤ 0.001. Scale bars represent 2 μm (**A,B,E**) and 1 μm (**F–H**).

The co-localization detected above was a strong evidence that FTY-720 induced heterotypic vesicular and mitochondrial fusion, such that we tested these initial observations by performing electron microscopy (EM) on control and FTY-720 treated cells. In electron micrographs of control untreated cells, relatively homogeneous electron-dense chromaffin granules were evident in the cytosol of the cells, and there was a lower density of mitochondria with a weaker electron density, identified by their shape and the presence of internal cristae (Fig. 4A). After incubating the cells for 15 min with FTY-720 (20 µM), the size of the electron-dense vesicles was clearly reduced (measuring more than 1,000 vesicles in 11 cells: Fig. 4B), with a 50% fall in their mean circular area (Fig. 4C). In addition, there were 50% fewer vesicles in the cytosol of the treated chromaffin cells (n = 11 cells, P < 0.001: Fig. 4C).

In parallel with the changes detected in the size and number of chromaffin vesicles, we also found that  FTY-720 altered the shape and area of the mitochondria (Fig. 4B), which became rounder (Fig. 4D) in conjunction with a four–fold increase in their area (Fig. 4D). Consequently, the EM images supported the data gathered by confocal and TIRF microscopy, proving the simultaneity of the changes observed in chromaffin vesicles and mitochondria when cells were treated with FTY-720.

In the same EM images, some of the events leading to the eventual fusion of organelles were evident. For example, the initial phase of vesicular homotypic fusion was observed (Fig. 4E), with the formation of vesicular contacts between two (Fig. 4F, second row) or three vesicles (third row). Subsequently, there was a redistribution of the dense core of the granules, suggesting a partial loss of the vesicular content that may even reach total release. Following the destiny of these multivesicular fusions, they commonly seemed to reflect complete fusion with mitochondria (Fig. 4G, second row), involving the fusion of 3 to 5 vesicles per mitochondria and the internalization of the vesicular content in the mixed organelle, without any loss of its compact aspect (Fig. 4G,H, second row). In other images, heterotypic fusion of individual chromaffin vesicles and mitochondria was evident as a possible early event that did not require the prior homotypic fusion of vesicles (Fig. 4H, second row).

After the formation of these mixed organelles, there was evidence of the fusion of these mixed organelles in other images (Fig. 4G, third and fourth row). As such, “giant” round organelles with many characteristics of mitochondria (such as the internal cristae) were ultimately formed, incorporating the dense core of several chromaffin vesicles (Fig. 4G,H, fourth and fifth rows).

### The fusogenic properties of FTY-720 are mediated by SNAREs, driving the changes in vesicles and mitochondria

**Figure 5:**
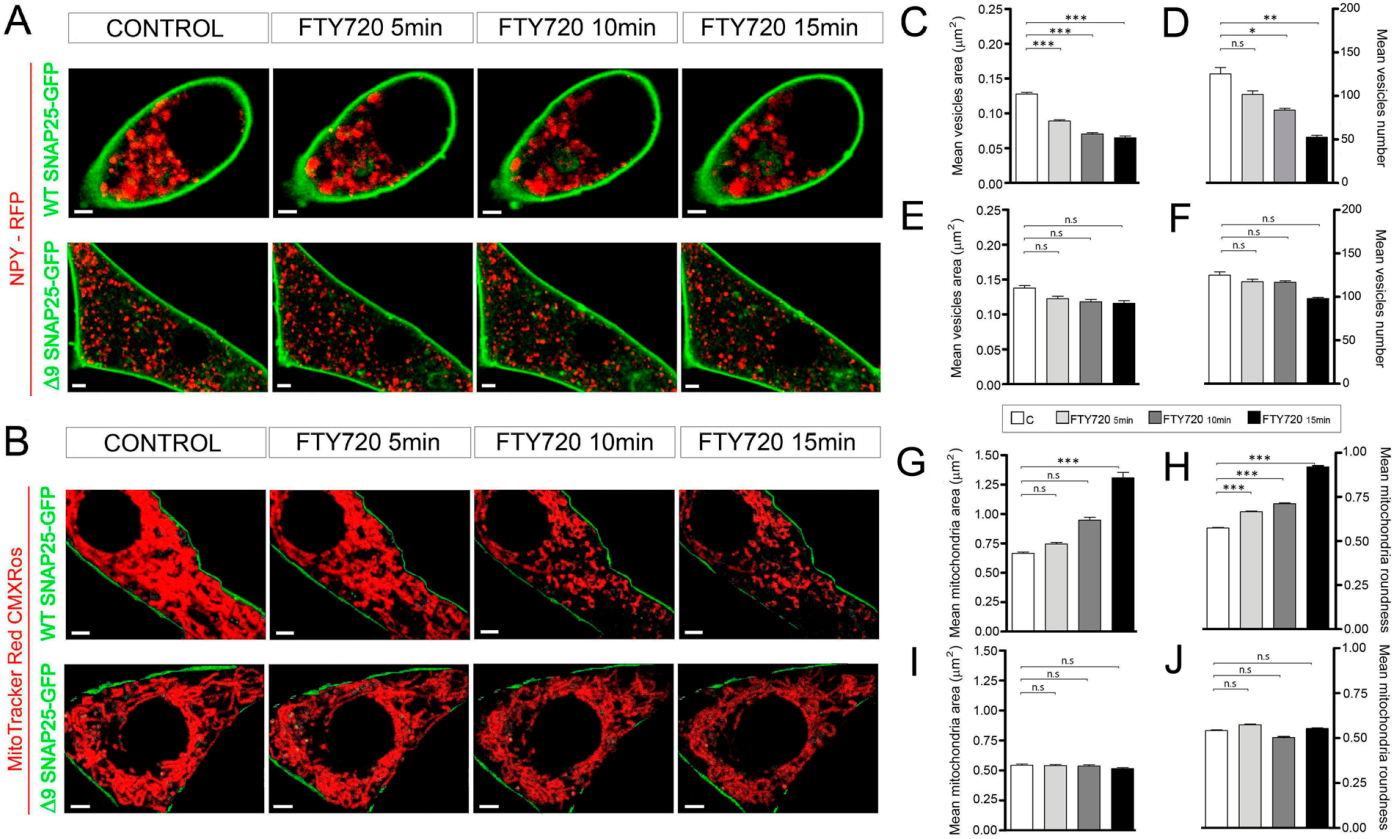
SNAP-25 is involved in organelle fusion induced by FTY-720. (**A,B**) Time-lapse confocal fluorescence microscopy images from representative cultured chromaffin cells expressing WT and the truncated form GFP-SNAP-25 ∆9 (green), combined with RFP-NPY (red) expression to show vesicles (**A**) and MitoTracker Red CMXRos labeling to detect mitochondria (**B**), in control cells and after FTY-720 treatment (5, 10 and 15 min). (**C,D**) Mean ± SEM values of vesicular area (**C**) and number (**D**) for WT SNAP-25 cells (N = 5 cells: N_control_ = 686, N_FTY720 5min_ = 507, N_FTY720 10min_ = 419, N_FTY720 15min_ = 263 vesicles). (**E-F**) Mean ± SEM values of vesicular area (**E**) and number (**F**) for ∆9 SNAP-25 cells (N = 5 cells: N_control_ = 601, N_FTY720 5min_ = 587, N_FTY720 10min_ = 584, N_FTY720 15min_ = 490 vesicles). (**G,H**) Mean ± SEM values of mitochondrial area (**G**) and roundness (**H**) for WT SNAP-25 cells (N = 5 cells: N_control_ = 665, N_FTY720 5min_ = 612, N_FTY720 10min_ = 420, N_FTY720 15min_ = 341 mitochondria). (**I,J**) Mean ± SEM values of mitochondria area (**I**) and roundness (**J**) for ∆9 SNAP-25 cells (N = 5 cells: N_control_ = 579, N_FTY720 5min_ = 582, N_FTY720 10min_ = 557, N_FTY720 15min_ = 497 mitochondria). Statistical significance was assessed with a Two-way ANOVA Test: n.s. non-significant (P ≥ 0.05), *P ≤ 0.05, **P ≤ 0.01, ***P ≤ 0.001. Scale bars represent 1 μm.

To test whether the alterations to chromaffin cells and mitochondria caused by FTY-720 are mediated by SNARE-dependent fusogenic properties, as demonstrated for exocytosis in different systems^13^, chromaffin cells were transfected with the wild-type SNAP-25 linked to GFP or a truncated SNAP-25 Δ9 that did not prevent SNARE complex formation but reduced secretory activity, mimicking botulinum neurotoxin type A activity^34,35^. In addition, some cells also expressed NPY-mRFP to visualize vesicles, testing the ability of FTY-720 to alter their size and number. Over-expression of the truncated SNAP-25 partially prevented (50% reduction) the formation of microvesicles (Fig. 5A,C,E) and the reduction in the chromaffin granule population provoked by FTY-720 (Fig. 5D,F). In addition, mitochondrial labelling of the cells (Fig. 5B) demonstrated that SNAP-25 Δ9 over-expression partially prevented the change in area (Fig. 5G,I) and in the roundness of mitochondria (Fig. 5H,J). Together, these results strongly suggest that a late phase of SNARE-mediated fusion is involved in the alterations suffered by these organelles as a result of FTY-720-enhancement of SNARE complex formation^13^.

### Mitochondrial alterations affect their redox activity and promote cell toxicity

**Figure 6:**
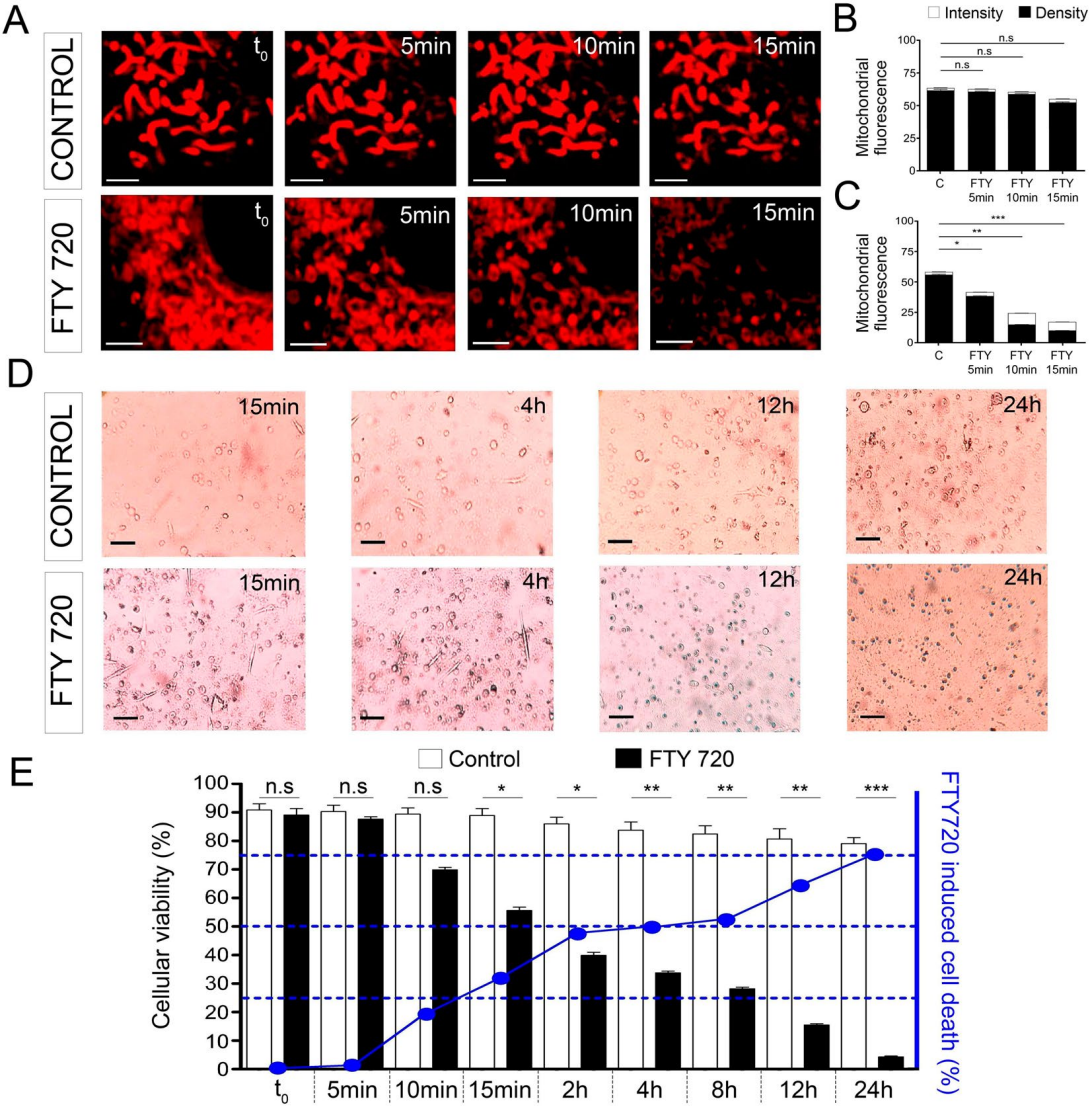
Altered mitochondrial membrane potential and reduced cell viability promoted by FTY-720. (**A**) Time-lapse confocal fluorescence microscopy images from a representative control and FTY-720 treated cultured chromaffin cell (t0, 5, 10 and 15 min) labeled with MitoTracker Red CMXRos to identify mitochondria. (**B,C**) Mean ± SEM mitochondrial membrane potential in terms of fluorescence intensity (white bars) and density (black bars) for control condition (**B**) (N_control_ = 5 cells; N_T0_ = 797, N_5min_ = 781, N_10min_ = 764, N_15min_ = 760 mitochondria) and FTY720 treatment (**C**) (N_FTY720_ = 5 cells; N_TO_ = 804, N_5min_ = 621, N_10min_ = 424, N_15min_ = 350 mitochondria). (**D**) Time-lapse optical microscopy images from representative chromaffin cell cultures under control conditions and after FTY-720 treatment for 15 min evaluated at different times: 15 min, 4 h, 12 h, and 24 h. (**E**) Mean ± SEM of the viability of control (white bars) and FTY-720 treated (black bars) cells evaluated at distinct times (N_t0_ = 467 (**C**), 488 (FTY); N_5min_ = 503 (**C**), 453 (FTY); N_10min_ = 497 (**C**), 468 (FTY); N_15min_ = 510 (**C**), 505 (FTY); N_1h_ = 477 (**C**), 464 (FTY); N_2h_ = 396 (**C**), 478 (FTY); N_4h_ = 470 (**C**), 482 (FTY); N_8h_ = 567 (**C**), 538 (FTY); N_12h_ = 477 (**C**), 445 (FTY); N_24h_ = 455 (**C**), 476 (FTY) cells). Statistical significance was assessed with a Mann-Whitney Test: n.s. non-significant, (P ≥ 0.05), *P ≤ 0.05, **P ≤ 0.01, ***P ≤ 0.001. Scale bars represent 1 μm (**A**) and 15 μm (**B**).

To assess the functional consequences of the alterations to the organelles produced by FTY-720, we evaluated its effects on the mitochondrial redox potential by measuring the intensity of mitotracker red fluorescence that is sensitive to this parameter^36^. There was an obvious reduction in the Mitotracker red labelling in cells incubated with FTY-720 (20 µM), both in terms of intensity and fluorescence density (Fig. 6A). This reduction in intensity reached around a 70% decrease following a 15 min incubation with FTY-720 (Fig. 6C), indicative of a strong effect on the mitochondrial redox potential.

Impaired mitochondrial function will almost certainly affect cell survival and therefore, we studied the maintenance of cell integrity by Trypan Blue staining (Fig. 6B). As expected, after a 10 min incubation with FTY-720 around 20% of cells took up this dye, indicative of the disruption of their cell membrane (Fig. 6D). Moreover, after a 15 min incubation with FTY-720 (as used in most experiments), followed by a 2 h recovery in fresh medium, we detected 50% cell death relative to the controls. Finally, 24 h after this 15 min incubation even more cell mortality was evident, affecting 80% of the cell population. Thus, it appeared that the rapid and dramatic alterations to mitochondrial morphology caused by FTY-720 via their heterotypic fusion with vesicles was accompanied by a dissipation of their redox potential, which led ultimately to largescale mortality of the cultured cells.

## Discussion

### FTY-720 a sphingomimetic drug influencing multiple physiological processes in the nervous system

FTY-720, also known as Fingolimod, is a structural analogue of sphingosine with immunosupressant properties^22^ that has been approved and is used extensively to treat relapsing remitting MS^12,37^. Its activity is related to the capacity of sphingosine-1P to interact with receptors mediating lymphocyte homing, thereby avoiding immunosupression^38^. Nevertheless, in the last 5 years evidence has accumulated that this compound also influences a variety of processes, including neuronal gene expression, axon growth and degeneration^39^. These effects may not only be associated with the neuroprotective benefits of this drug^40^ but also, its therapeutic effects in ischemia, excitotoxicity and memory recovery^41–43^. Nevertheless, this drug could also negatively affect neurons, inducing apoptosis by altering calcium signalling and mitochondrial membrane potentials, effects that could be useful to sensitize neuroblastoma cells to anti-.neoplastic drugs^19^.

In terms of the mechanisms that might underlie such a variety of neuronal effects, we recently demonstrated that FTY-720 mimics sphingosine in its activation of synaptobrevin to promote SNARE complex formation, enhancing exocytosis in neuronal and neuroendocrine models at concentrations around 10–20 µM, similar to that found for sphingosine^13^. Here, we show that FTY-720 enhances quantal vesicle release, consistent with the results obtained in rat hippocampal neurons in culture^13^. However, rather than a simple increase in the release of neurotransmitter per event, we observed the formation and fusion of microvesicles, events that ultimately provoke an increase in the mean exocytotic release. These complex effects on exocytosis seem to agree well with the effects of sphingosine, enhancing release from microvesicles and full chromaffin granules in bovine^23^ and rat chromaffin cells^24^. Interestingly, this effect was also found in lactotrophs^44^, suggesting that the size of vesicles was a major factor influencing the effect of sphingosine, favouring the “kiss and run” fusion of microvesicles and the full collapse of larger dense vesicles.

Our confocal and EM studies clearly show that microvesicles are produced rapidly in the cytosol following FTY-720 treatment, a process that is dependent on SNARE activity. Indeed, some of these vesicles undergo homotypic fusion that could lead to exocytotic fusion, releasing distinct levels of catecholamines, as detected by single cell amperometry.

### FTY causes heterotypic fusion of secretory granules and mitochondria

In searching for possible changes in chromaffin vesicle size to explain the variation in quantal exocytosis, we found important alterations to mitochondrial size and aspect that reflect their heterotypic fusion. Hence, this is the first report of heterotypic fusion linked to the action of an approved drug. The mechanisms underlying this organelle fusion involve SNARE proteins present in the membrane of chromaffin granules. The observation of homotypic granular fusion concomitant with heterotypic fusion during the initial phase of FTY-720 treatment strongly suggests the SNAREs are incorporated into the mitochondrial membranes, in stark contrast to their normal distribution. Subsequently, mitochondria also undergo homotypic fusion to generate giant altered forms of these organelles that include several dense granule cores (normally between 2 and 5). Since this is a late event, it might be related to the incorporation of SNAREs during heterotypic fusion.

### A novel mechanism of action of FTY-720 causing mitochondrial impairment and cell death

**Figure 7:**
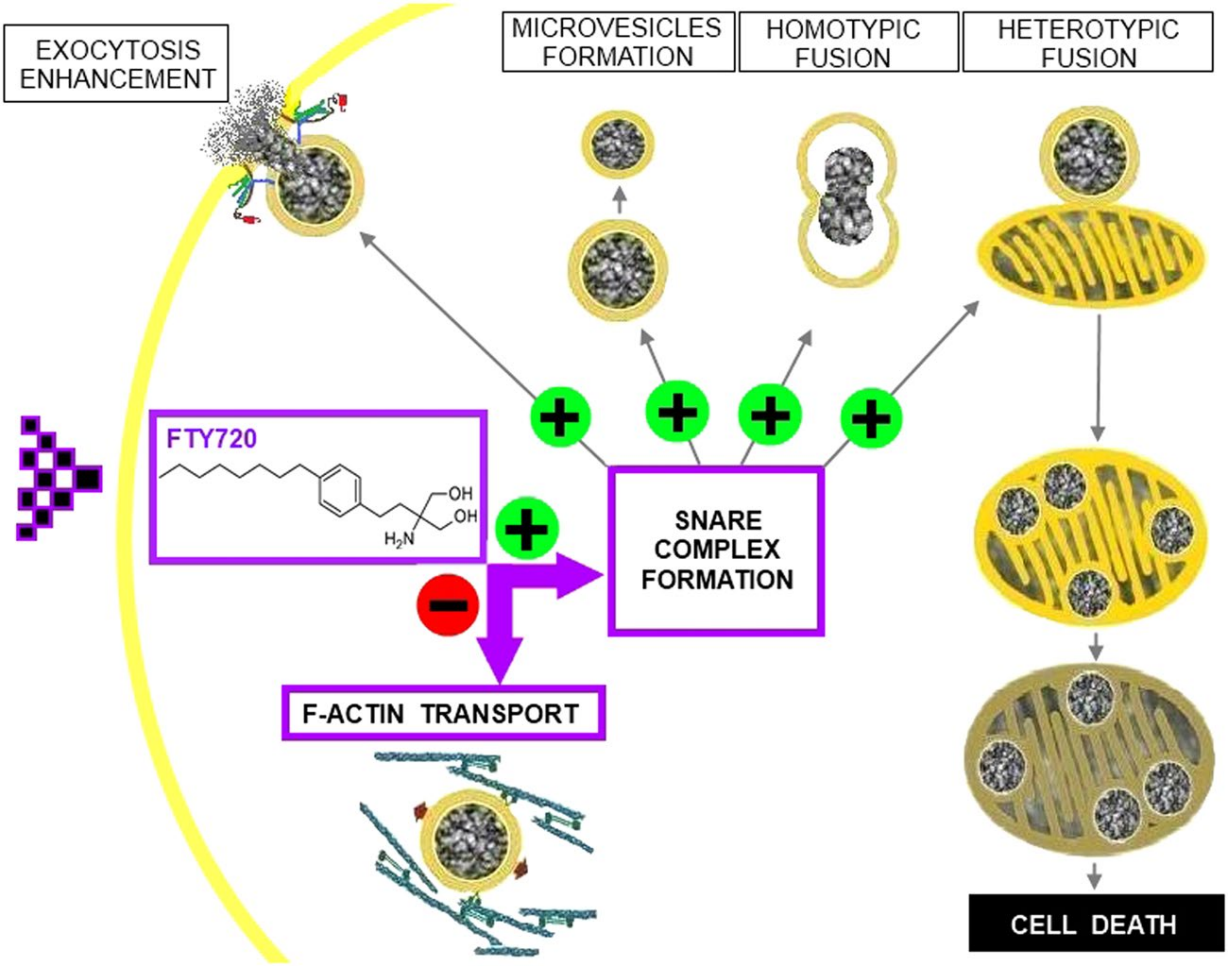
A new mechanism of action for FTY-720 affecting neuroendocrine function and cell death. FTY-720 accumulates in the cell cytoplasm where it can promote SNARE complex assembly and reduce F-actin mobility, thereby limiting vesicle transport to the plasma membrane for secretion. SNARE complex enhancement mediates intracellular vesicle fission. Vesicles could fuse among themselves (homotypic fusion) or with mitochondria (heterotypic fusion). Mixed organelles cannot maintain their membrane potential, leading to cell death.+Indicates potentiation and − reduction.

The data presented here clearly support a new mechanism that explains the effects of FTY-720 on neuronal and neuroendocrine cells, and that is driven by the enhanced fusogenic activity of SNAREs induced by micromolar concentrations of this drug^13^. Accordingly, FTY-720 may diffuse across the plasma membrane to accumulate in the cytosol, where it could mimic the activity of sphingosine to promote SNARE complex formation, influencing synaptobrevin availability^13^. The formation of these fusogenic complexes enhances the exocytotic fusion of secretory vesicles, initially augmenting neuroendocrine secretion as occurs with sphingosine derivatives^10,23,24^. However, the concomitant inhibition of F-actin cytoskeleton mediated transport limits the availability of vesicles and their transport from internal pools, as demonstrated here and in accordance with data from astrocytes^28^. Thus, FTY-720 has a complex effect on exocytosis, promoting the release of ready releasable pools of vesicles but impairing the recruitment of other pools from the cell’s interior (see scheme in Fig. 7).

Interestingly, SNARE activity is also involved in the formation of microvesicles by membrane fission and homotypic fusion of granules in the cytosol (Fig. 7). The enhanced fusogenic properties of vesicles also leads to the heterotypic fusion of vesicles with mitochondria and finally, to the homotypic fusion of altered mitochondria. As evident in EM images, the alterations to mitochondrial structure are so dramatic as to collapse the mitochondrial membrane potential, which probably provokes rapid apoptosis and cell death (Fig. 7). Similar alterations to the redox potential of mitochondria provoked by FTY-720 have been seen in neuroblastoma cells, suggesting this compound induces apoptosis of these tumour cells through various signalling pathways^19^. In conclusion, as far as we know this is the first report of drug induced heterotypic fusion of organelles that induces cell death. This effect must be taken in account when interpreting the effects of FTY-720, compromising the viability of target cells and potentially representing a valuable tool to attack populations of tumour cells of neuronal origin.

## Methods

### Chromaffin cell preparation and culture

Chromaffin cells were isolated from bovine adrenal glands by collagenase digestion, and they were further separated from the debris and erythrocytes by centrifugation on Percoll gradients, as described elsewhere^45–47^. The cells were maintained as monolayer cultures in Dulbecco’s modified Eagle’s medium (DMEM) supplemented with 10% foetal calf serum, 10 μM cytosine arabinoside, 10 μM 5-fluoro-2′-deoxyuridine, 50 IU/ml penicillin and 50 μg/ml streptomycin. The cells were harvested and plated at a density of 150,000 cells/cm^2^ in 35 mm Petri dishes (Costar), and they were used between the third and sixth day after plating.

### Amperometric determination of exocytosis

To study secretory activity from control non-transfected cells and those expressing the different constructs, the culture medium was replaced by Krebs/HEPES (K/H) basal solution the pH of which was adjusted to 7.4 using NaOH (in mM): NaCl 134, KCl 4.7, KH_2_PO_4_ 1.2, MgCl_2_ 1.2, CaCl_2_ 2.5, Glucose 11, and Hepes 15. Carbon-fibre electrodes insulated with polypropylene and with 14 µm diameter tips were used to monitor catecholamine release from individual chromaffin granules in cells under superfusion^26^. The electrodes were positioned in close apposition to the cell surface using high precision hydraulic micromanipulation, assessing cell membrane deformation with an Axiovert 135 inverted-stage microscope (Zeiss, Oberkochen, Germany) carrying Hoffman optics (Modulation Optics, Greenvale, NY). Electrical connection was accomplished with mercury and an amperometric potential of +650 mV was applied against an Ag/AgCl bath reference electrode using an Axopatch 200 A amplifier (Axon Instruments Inc. Foster City, CA). The current product of catecholamine oxidation was digitized with an A/D converter and recorded at 400 μs/point using the Clampex software (Axon) running on a PC. Experiments were performed in cells stimulated by superfusion with a depolarizing 59 mM high potassium solution (obtained by isosmotically replacing NaCl with KCl), applied through a valve-controlled puffer tip commanded by the acquisition software and located near the cells studied. A curve fitted to the non-linear models provided or implemented in the software (Igor Pro and Graphpad Prism) was used to analyse the data. Individual spike characteristics were studied using the aforementioned Quanta program, allowing for peak detection, integration and kinetic parameter calculations. After acquisition at 2 kHz, only well- defined narrow peaks with an amplitude higher than 5 pA were used to build event histograms. This ensured that the vesicle fusions analysed were produced in the proximity of the electrode and thus, complete oxidation of the released catecholamines was accomplished. Cell-to-cell variation was alleviated by using the same electrodes to measure the control cells and those exposed to FTY-720 (20 µM) for 15 min (Sigma Aldrich, SML0700, Lot#056M4724V), as indicated elsewhere^13^.

### Confocal fluorescence microscopy of chromaffin granules, mitochondria, SNAP-25 and secretion zones

Plasmids were expressed as described elsewhere^48,49^, using RFP-NPY (red) to identify dense-core vesicles and EGFP-SNAP25 (green) to study both the WT and ∆9 SNAP-25 protein. The Amaxa basic nucleofector kit was used to transfect primary mammalian neuronal cells according to the manufacturer’s instructions (program 0–005, Amaxa GmbH, Koehl, Germany). Moreover, cells were incubated for 15 min with MitoTracker Green FM (0.1 μM: Invitrogen-Molecular Probes ref M7514, Lot#1391-10) and MitoTracker Red CMXRos (0.2 μM: Invitrogen-Molecular Probes ref. M7512, Lot#1644639) to visualize mitochondria, as described previously^30^. MitoTracker labeling was used in transfected (two days post-transfection) and non-transfected cells, and combined with Alexa Fluor 488 (green) to locate active sites of secretion at the plasma membrane by visualizing DBH protein patches. All fluorescent labelling was performed prior to FTY-720 treatment (20 μM: Sigma Aldrich, SML0700, Lot#056M4724V), except the immunolabeling.

For immunolabeling, cells were first incubated with Mitotracker Red (CMXRos), as described above, and they were then treated with FTY-720 for 5 or 15 min prior to studying their basal state and that after stimulation by depolarization with a 59 mM KCl solution for 30 s at room temperature (21–22 °C). Secretion was stopped by lowering the temperature with ice-cold buffer and the sites of secretion were labelled by immunocytochemistry using an anti-DBH rabbit antiserum (1/500: Merck-Millipore, ref AB1585), followed by an Alexa Fluor 488 coupled donkey anti-rabbit IgG (H + L) secondary antibody (1/200: Molecular Probes, ref A21206), again in ice-cold buffer to prevent endocytosis.

Vesicles, mitochondria, SNAP-25 proteins and secretory sites were studied in confocal fluorescence images from equatorial cellular sections, all obtained with an Olympus Fluoview FV300 confocal laser system mounted on an IX-71 inverted microscope incorporating a 100X PLAN-Apo oil-immersion objective with 1.45 NA. Excitation was achieved with argon and helium-neon visible light lasers, and dual fluorescence was registered by sequential acquisition using a 488 nm argon ion (40 mW) to excite EGFP, MitoTracker green and Alexa 488, and with a 543 nm He/Ne 10 mW for RFP and MitoTracker Red CMXRos. A coupled perfusion system was used for FTY-720 treatment, administered at room temperature (22–25 °C) during time-lapse recordings for 15 min.

### TIRF microscopy of chromaffin granules

We used transfection of the RFP-NPY plasmid, as described above, to label dense-core vesicles, and two days after transfection live chromaffin cells were recorded by TIRF microscopy after a 5 min incubation with K/H solution followed by 15 min with FTY-720 (20 μM: Sigma Aldrich, SML0700, Lot#056M4724V). All these solutions were applied using the perfusion system at room temperature (22–25 °C).

A through-the-lens TIRFM system was configured on the Olympus IX-71 inverted microscope using a 100× PlanApo 1.45 NA Olympus TIRFM objective^33^. Laser illumination (543 nm He/Ne 10 mW: MellesGriot, Carlsbad, CA, USA) was selected using an Olympus TIRFM IX2-RFAEVA combined system, modifying the angle of laser incidence. In these experiments, laser intensity was kept low to prevent light-induced fusion (2–4% of the maximal intensity)^50^. TIRFM calibration was performed using 100-nm fluorescent beads (Molecular Probes, Invitrogen Detection Technologies, Carlsbad, CA, USA). The depth of penetration for the evanescent field was estimated to be around 200 nm (1/e depth of 180 ± 16 nm), mainly permitting the visualization of the static beads adhered to the coverslip. Fluorescence emission was acquired at a rate of 5 s per frame using an Electron Multiplier charge-coupled device cooled camera (C9100-02 model, Hamamatsu photonics, Japan) and the data was stored on an IBM compatible PC.

### Transmission electron microscopy

Bovine chromaffin cell control pellets and pellets pre-treated for 15 min with FTY-720 (20 μM: Sigma Aldrich, SML0700, Lot#056M4724V) were fixed for 2 h at 4 °C with 2.5% glutaraldehyde in 0.2 M cacodylate buffer [pH 7.0]. All the pellets were then washed overnight at 4 °C in a solution of 0.2 M cacodylate buffer, sucrose and distilled water and after post-fixing for 2 h at 4 °C with 1% osmium tetroxide in 0.2 M cacodylate buffer, and extensive washing with distilled water, the samples were stained for 1 h at 4 °C with 2% aqueous uranyl acetate. Subsequently, the samples were washed again, dehydrated through an ethanol series (30, 50, 70, 80, 96 and 99%: 15 min in each) and incubated twice for 15 min with propylene oxide at room temperature. Finally, the cell pellets were embedded in epoxy resin and ultra-thin sections (70 nm) were obtained on a Leica UC6 ultramicrotome and transferred to copper grids (200 mesh). After staining with uranyl acetate for 5 min and lead citrate for 1 min, the ultrathin sections were analyzed on a JEOL 1011 80 kv transmission electron microscope, with a Gatan BioScam mod 792 digital camera to capture the images.

### FTY-720 toxicity on cultured chromaffin cells

We used cell monolayer cultures incubated with supplemented DMEM (see above) and trypan blue solution (1:5: Sigma Aldrich, T6146) cultured for 24 h and evaluated at: t_0_, 5 min, 10 min, 15 min, 1 h, 2 h, 4 h, 8 h, 12 h and 24 h. We prepared control cultures and others that were treated with FTY-270 (20 μM; Sigma Aldrich, SML0700, Lot#056M4724V) for 5, 10 and 15 min. In some cases, after the 15 min FTY-720 treatment the culture medium was replaced by fresh DMEM-Trypan Blue to remove the FTY-720 and the long-term toxicity produced was studied. This evaluation consisted of quantifying cell death (blue cells that incorporated trypan blue through their compromised membrane) and live cells, comparing the control and FTY-720 treated cells at each time points. Cultures were assessed and the cells counted at each time point using an optical microscope coupled to a camera. The numbers of dead and live cells were used to calculate the cell viability (CV, %) for each condition and time, as follows:

$$CV\, \% =[Number\,of\,live\,cells/(Number\,of\,dead\,cells+Number\,of\,live\,cells)]\ast 100$$

### Statistical analysis

The data are presented as the mean ± SEM (standard error of the mean) and the statistical significance was determined using different tests based on the number of evaluation conditions, the influencing factors and the parametric nature of the data (GraphPad Prism® 4.0, GraphPad Software Inc., San Diego, CA). A *Student’s T-test* was used to evaluate parametric data with a single influencing factor, and a *Mann-Whitney U-Test* for two evaluation conditions with a single influencing factor (not assuming a Gaussian distribution). A *Kruskal-Wallis Test* was used for more than two evaluation conditions with a single influencing factor (not assuming a Gaussian distribution) and a *Two way ANOVA Test* was used for parametric data where there were two or more evaluation conditions with two influencing factors. The statistical significances are included in the graphs as: n.s, non-significant (P ≥ 0.05); *P ≤ 0.05; **P ≤ 0.01; ***P ≤ 0.001. *“N”* values reflect the number of cells and organelles analyzed (vesicles and mitochondria) in each condition, and all are also included in the figure legends.

### Ethics statement

Adrenal glands were obtained from an industrial slaughterhouse (Matadero de Orihuela SA) that is subject to strict regulations laid down by the Spanish Ministries of Agriculture, Industry and Health, and in accordance with EC guidelines. All the protocols used in this study were approved by the “Organo Evaluador de Proyecto” at the University Miguel Hernández, the office in charge of overseeing the ethical issues associated with animal care and experimentation at our institute.
